# Applications and Pharmacological Properties of Cactus Pear (*Opuntia* spp.) Peel: A Review

**DOI:** 10.3390/life12111903

**Published:** 2022-11-16

**Authors:** Salvador Manzur-Valdespino, José Arias-Rico, Esther Ramírez-Moreno, María de Cortes Sánchez-Mata, Osmar Antonio Jaramillo-Morales, Julieta Angel-García, Quinatzin Yadira Zafra-Rojas, Rosario Barrera-Gálvez, Nelly del Socorro Cruz-Cansino

**Affiliations:** 1 Área Académica de Nutrición, Instituto de Ciencias de la Salud, Universidad Autónoma del Estado Hidalgo, Circuito Ex Hacienda La Concepción S/N, Carretera Pachuca-Actopan, San Agustín Tlaxiaca 42160, Mexico; 2 Área Académica de Enfermería, Instituto de Ciencias de la Salud, Universidad Autónoma del Estado Hidalgo, Circuito Ex Hacienda La Concepción S/N, Carretera Pachuca-Actopan, San Agustín Tlaxiaca 42160, Mexico; 3 Department of Nutrition and Food Sciences, Pharmacy Faculty, Universidad Complutense de Madrid, Plaza de Ramón y Cajal s/n, E-28040 Madrid, Spain; 4 Nursing and Obstetrics Department, Life Sciences Division, Campus Irapuato-Salamanca, University of Guanajuato, Ex Hacienda El Copal, Km. 9 Carretera Irapuato-Silao, A.P 311, Irapuato 36500, Guanajuato, Mexico

**Keywords:** waste, by-product, composition, bioactive compounds, health benefits

## Abstract

Nowadays, there is a growing interest in the exploitation of by-products from fruits and vegetables, generated from industrial processing or human feeding. Residues of popularly consumed fruits such as orange, lemon, banana, pomegranate, among others, have been widely described and studied; however, cactus pear (*Opuntia* spp.) residues, as a locally consumed product, have been forgotten. The whole fruit can be divided into the edible portion (pulp) and the non-edible portion (seeds and peel). Several studies mainly focus on the characteristics of the edible portion or in the whole fruit, ignoring by-products such as peels, which are rich in compounds such as phenols, flavonoids and dietary fiber; they have also been proposed as an alternative source of lipids, carbohydrates and natural colorants. Some uses of the peel have been reported as a food additives, food supplements, as a source of pectins and for wastewater treatment; however, there have not been any deep investigations of the characteristics and potential uses of the cactus pear peel (CPP). The aim of the present paper is to provide an overview of the current research on CPP. CPP has many bio-active compounds that may provide health benefits and may also be useful in pharmaceutical, food and manufacturing industries; however, greater research is needed in order to gain thorough knowledge of the possibilities of this by-product.

## 1. Introduction

Cactus pear is the fruit of the nopal cactus, is native to the arid and semi-arid regions of Mexico and Mesoamerica and has spread to many regions [1,2] This fruit is commonly known as cactus pera fruit, prickly pear, tuna (Mexico), higo (Colombia) higo chumbo (Spain), fico d’India, figue de barbarie (France), among others. There are some countries where there is significant production of this fruit, such as Italy, which is the most important producer in the Mediterranean area, and on the African continent, it is produced in the Cape region and in South Africa; in countries such as Israel, Chile and Argentina, it is produced on a small scale and it is also possible to find it in some plantations in Brazil, Colombia, Peru, Spain, Greece and Turkey [3]. However, Mexico is the main producer worldwide and cactus pear cultivation is considered highly profitable, because in optimal conditions, the production is 40 tons per hectare by year [2,4]. In Mexico, an area of 48,000 ha is dedicated to its cultivation, in which 352,000 tons by year are produced, through the participation of around 20,000 producers [2,5]. The main producing regions in Mexico are the southern region (Puebla and Oaxaca), the central region (Estado de Mexico and Hidalgo) and the north-central region (Guanajuato, Jalisco, Aguascalientes, San Luis Potosí and Zacatecas) [2,6].

It is estimated that the consumption per capita is only around 3.7 kg/year, since it is a seasonal fruit and is only available a few months per year [7]. The fruit is very popular, and it is mainly consumed fresh, although it is also processed in products manufactured on a small scale or in an artisanal way, and can be found in jams, yogurts, juices or candies [8]. Because of how it is consumed and processed, only the edible portion is used, generating a large amount of residue between peels and seeds. The non-edible portion known as the peel comprises two fractions, the mesocarp and the pericarp, and depending on the variety, it may represent between 33 to 55% of the total weight. It is usually discarded as by-product and may represent a problem due to waste management issues [9,10,11]; therefore, different alternatives have been sought for its use, revealing that is an inexpensive source of many nutrients, such as minerals [12], aminoacids [13], polyunsaturated fatty acids [14] and carbohydrates (which have been applied as a source of fiber, sweeteners and pectins for food applications) [15].

In addition, several studies have shown that cactus pear by-products are rich in bioactive compounds [16] such as phenolics, flavonoids, pigments, fibers, polysaccharides and fatty acids [8]. They can provide many health benefits such as inhibition and protection against free radicals [17], cytotoxic activity against some cancer cell lines [18] as well as the reduction of atherosclerosis and glycaemia [19]. However, many studies have been performed by using the pear peel for multiple purposes such as for pigment extraction [20] a preservative for margarine [21], as snacks [22], a dietary supplement with hypoglycemic properties [23] and in wastewater treatment [24].

The present review describes the nutritional characterization of cactus pear peel as a promissing source in pharmaceutical, food, textile and wastewater industries.

## 2. Information Sources and Search Strategies

Scientific databases such as PubMed, Research Gate and Science Direct were used in the literature search, using the following search key words: *Opuntia* spp., Cactus pear and peel; as filters to search all fields, the words: composition, bioactive compounds, applications and health benefits were used. From the articles resulting from the search, the abstracts were carefully read, and relevant studies were selected and reviewed.

### Study Eligibility Criteria

**Figure 1:**
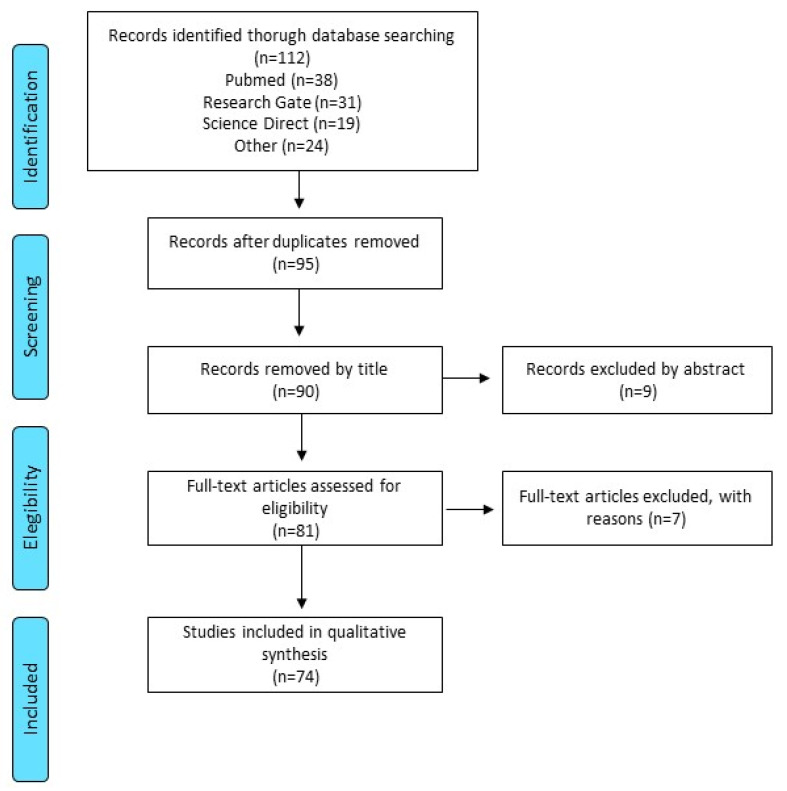
PRISMA flow-chart.

Original articles written in English and Spanish were included, which the pericarp and endocarp of the fruit were used or described; those in which the properties or effects of other by-products, such as the pulp or the seeds, were excluded. The process was carried out following the recommendations of the PRISMA Flow Diagram [25] as presented in Figure 1.

## 3. Cactus Pear Description and Structure

Cactus pear is the fruit of the *Opuntia* spp., which is a shrubby plant of the *Opuntia* genus and belongs to the Cactaceae family [26]. The fruit usually has a spherical or turbine shape, with elliptical or cylindrical variations. The color combinations such as bright yellow, green, red or purple are provided by pigments such as betanins and betaxanthins. The size is approximately 7 to 9 cm long and 5 to 6 cm wide, whereas the weight varies from 86 to 146 g. The peel has the same color of the pulp and has an umbilical zone from 50 to 70 “areolas”, with small spines from 3 to 10 mm [27] and the peel thickness is approximately 0.65 cm [28]. At the beginning of fruit development, the peel or skin predominates over the locular tissue, whereas as the fruit grows, the pulp proportion increases in comparison to the peel [29], and when the fruit matures, the peel still comprises the highest percentage [30]. 

The first function of the fruit peel is to protect the pulp from weather and the sun [31], and the peel also indicates the moment when the fruit is ripe through physical parameters, such as the change of color, shape, firmness, diameter and volume, which allow for knowing the optimal time for harvesting [29]. In the whole fresh fruit, the amount of peel may be of approximately 40%, and on a dry weight basis, it may represent 25% of the total weight [32].

Cactus pear peel (CPP) has been studied mainly for the extraction of starches, pectins and fiber. The peel cells are mainly composed of collenchyma and parenchyma cells, which are rich in pectic polysaccharides [33]. The organization and morphology of the different cell types that conform to the peel from the outside to the inside can be described as follows: the chlorenchyma cells are thin-walled epidermal cells that are rich in non-cellulosic components and have thick cell walls, and on the other hand, parenchyma cells show thin walls. Inside the tissues of the collenchyma and parenchyma, there are mucilaginous cells that act as a polysaccharide storage, which is common in succulent plants, and is related to the osmotic function for water molecule retention [32]. 

### 3.1. Physical Chemical Characteristics and Nutrient Composition 

The peel of the fruit shares some properties with the fruit; its physical characteristics give it very specific organoleptic properties, in addition to allowing for knowing the ripeness of the fruit. The acidity of CPP is 0.02 to 0.12% of citric acid, which gives the characteristic flavor of the fruit and is related to the pH, which is an indicator of the maturity of the fruit. CPP can reach values of 4.5–5.9, and in this sense, it could be considered a low acid by-product (pH > 4.5) [12,34,35]. Soluble solids content (°Brix) is a parameter used for the screening of the evolution and ripening of the fruit, correlating with the content of sugars through the refraction properties of the total soluble solids [36]. The peel °Brix varies from 6.16 to 15.00 depending on the state of maturity of the fruit at harvest time [12,35].

CPP also has nutritional properties of interest, which are summarized in Table 1. CPP is notorious for its high percentage of moisture and the low amount of lipids and proteins; however, it also has essential amino acids, which are described later.

Carbohydrates are the major component of CPP, which are rich in fibers and polysaccharides. It may change depending on the variety and color of the peel; for example, the peels of red cactus pear present high humidity and high soluble fiber content, whereas green fruit peels present a higher content of fat and insoluble fiber [11]. 

Since the demand for novel sources of quality protein have increased, the use of vegetables as an alternative source of protein has been proposed, especially those obtained from agro-industrial by-products. The use of cactus pear residue is available at low costs and can contribute to the generation of value-added protein, leading to environmental sustainability [39]. 

It is well known that the amino acids of CPP are linked to the betaxanthins, one of the main pigments of the cactus pear, which result from the conjugation of betalamic acid with protein or non-protein amino acids and biogenic amines. In CPP, sixteen betaxanthins have been identified, which include amino acids in their structure; they are arginine-betaxanthin, aspartic acid-betaxanthin, lysine-betaxanthin, proline-betaxanthin, serine-betaxanthin, tyramine-betaxanthin and threonine-betaxanthin [13].

Fruit peels could be considered as a promising source of essential fatty acids and fat-soluble antioxidants. According with Ramadan and Mörsel [14], around 36.8 g/kg (dry weight) of lipids have been found [14], and depending on the variety, the fatty acids that may be present in the peels are Lauric acid (C12:0), Myristic acid (C14:0),Palmitic acid (C16:0), Palmitoleic acid (C16:1), Stearic acid (C18:0), Oleic acid (C18:1), Linoleic acid (C18:2), Linolenic acid (C18:3) and Arachidonic acid (C20:0) [40]. From the recovered lipids, the unsaponifiable comprises 12.8% [14]. However, the amount of lipids is not only influenced by the color or variety, but is also affected by processing and storage conditions [12], since during the storage of oils and fats, lipid peroxidation takes place, affecting the nutritional and organoleptic properties, as the unsaturated nature of the fatty acids from cactus pears makes them highly susceptible to oxidation [9,11]. The fatty acids quantifications that are specifically found in the peel of the fruits of *Opuntia* spp. L. Mill are shown in Table 1. 

Mineral composition is highly influenced by the soil where the plant is grown and may vary from place to place, together with the variety of the fruit and the climate of that region. CPP is characterized by a high content of Mg and Ca [12] (Table 1). In this sense, the consumption of only 20 g of peel would cover 90% of the recommended daily intake (RDI) of magnesium and 20% of calcium for the general population [41]. Although, it is important to mention that calcium is not bio-accessible [10,11,42]. In addition, the presence of potassium is very important, since it helps to mitigate the negative effects of high sodium consumption on blood pressure [43], and manganese, zinc and copper are also present in the peels, being relevant because they are used for bone mineralization, muscle contraction, nerve stimulus transmission and act as a cofactor of many enzymes involved human metabolism [41,44,45]. 

As shown in Table 1, the peel contains mainly vitamin C, B3, B6 and B9 [46], whereas other vitamins, such as, thiamine or riboflavin, are found in trace amounts [47,48].

Ascorbic acid is one of the antioxidant agents found in abundance in the peels in comparison with the pulp [49], ranging from 46.40 to 86.28 mg AAE (Ascorbic Acid Equivalent)/100 g [50], and there are variations in the content depending on the color of the fruit; the highest content of ascorbic acid has been found in pink peels, followed by the orange ones and, lastly, the red varieties [50].

### 3.2. Bioactive Compounds

In some varieties of cactus pear fruit, the total phenolic content (TPC) as well as ascorbic acid is higher in the peel [51,52,53], mainly in red peel fruits [54], and a high amount is associated with the matrix of the dietary fiber [55]. Amounts up to 1534 mg GAE (Gallic Acid Equivalent)/100 g (fresh weight) can be found in the peels. The amount found depends on the cultivar, season and soil properties [49,53], fruit maturity and climate [53,56], as seen in Table 2. 

Purple peels have high concentrations of betalains, which are water-soluble, natural pigment derivatives, which yield a variety of colors, from red-violet (betacyanins) to yellow-orange (betaxanthins) [58,59], whereas green peel varieties have the lowest concentrations of these pigments.

The lipids from CPP present high levels of β-carotene [14]; the main carotenoids of the peel (representing about 80%) are lutein, β-carotene and violaxanthin [50]. In the different varieties of cactus pear (red, yellow, orange and green), the presence of carotenoids varies; however, the presence of norbixin, antheraxanthin, astaxanthin, canthaxanthin and ζ-carotene is consistent [57,60,61,62,63]. 

There is little research about the safety of oral ingestion of fruit peels, especially in the presence of pesticides, and there are minimal amounts of pesticides (such as malathion, chlorpyrifos, permethrin, diazinon, dimethoate, spinosad and abamectin) and heavy metals (copper, chromium, arsenic, cadmium, lead, and selenium). However, these are under the maximum limits of toxic residues established by the North American Free Trade Agreement (NAFTA), so that phytotoxic elements do not trigger health risks [8,11]. 

### 3.3. Potential Uses, Applications and Health Benefits

Many authors have focused their interest on this fruit, valuing it as a functional food because of its high fiber and secondary metabolites (polyphenols, betaxanthins, organic acids, among others) that confer properties such as having an anti-inflammatory effect, are lipid lowering, have a hypoglycemic effect, among others [64,65,66,67,68,69,70,71,72,73]. In these diseases, the chronic use of conventional anti-inflammatory drugs may lead to some adverse effects. For that reason, the anti-inflammatory activity of some natural compounds present in food products or herbal drugs may be valued as adjuvants to relieve the symptoms of these diseases [67].

#### 3.3.1. Digestive System

In an animal model of colonic inflammation caused by irradiation, it was observed that by applying a pretreatment with *Opuntia* spp. peel extract in rats, a prophylactic effect against the damage is produced in the colonic tissue, decreasing inflammation markers, as well as increasing intrinsic anti-inflammatory agents [73] because of the high content of phenolic and flavonoids that are linked to the by-products of the cactus pear [11]. Therefore, *Opuntia* spp. fruit peel extract could have some potential to improve colonic inflammation processes [73].

There are also other digestive issues such as constipation that is related to a deficient intake of fiber. This component plays an important role in human health since it is associated with prevention and the treatment of diseases such as colitis, colon cancer and high cholesterol levels (36). *Opuntia* ssp.peel is considered a good source of dietary fiber because 40.8% of the dry weight is fiber [33], and the recommended daily intake of dietary fiber in adults must be >25 g/day [74]; therefore, the daily intake could be easily covered with 62 g of dried CPP [31]. The beneficial effects of fibers in human health are widely known through the effects on the digestive system, helping to relieve constipation, increasing the bulk and softness of the feces on the intestine, accelerating the pass through the bowel and easing the evacuation [74]. In addition, dietary fibers have some functional properties such as an oil and water retention capacity, and which have lipid-lowering and anti-constipation effects, respectively [75]. 

It has been demonstrated in studies in vivo with rats that the consumption of cactus pear residues favors the growth of beneficial bacteria in the gut. In a study with rats fed with CPP flour, it was observed that the growth of lactic acid bacteria and bifidobacteria is promoted [76] because of the combination of non-digestible sugars with different dietary fibers [77].

#### 3.3.2. Antimicrobial, Antifungal, Antiviral and Insecticidal Activities

*Opuntia* spp. peel extract is considered a promising source of new natural antibacterial agents against some microbes, and peel extracts have been proven to have significant antimicrobial activity, which can vary according to the type of extract. The ethanolic extract of the peel has high activity against some microbes, even higher than the activity presented by the pulp extracts, showing an increase in the inhibition zone against *Staphylococcus aureus* and *Escherichia coli*. The peel extract also has antifungal activity against *Candida albicans* and has antibacterial activity against Gram-positive and Gram-negative bacteria because of the presence of several potent bioactive components such as sterols, tannins, alkaloids and other phenolic compounds [46].

Different solvents were used on the CPP in order to extract the bioactive compounds, and to evaluate the inhibitory effect against pathogens that cause pneumonia. Extractions with ethyl acetate (EtOAc) show the highest effect against microbes such as *Streptococcus pneumoniae*, *Stenotrophomonas maltophilia* and *Klebsiella pneumoniae*, among others. Furthermore, the isolated compound with the highest antimicrobial activity is Quercetin 5,4’-dimethyl ether [78]. Extracts of CPP have shown greater antimicrobial activity against Gram-positive bacteria than Gram-negative bacteria and antimicrobial activity against S. typhimurium and Bacillus subtilis [79] has been demonstrated. In addition, *Opuntia* ssp. peel extracts showed potential antiviral activity against H5N1 and rotavirus [80]. Concerning insecticidal activity, prickly pear peel waste has shown larvicidal activity as well as a decrease in the fecundity and hatchability of the *C. pipiens* mosquito [81]; however, more studies are needed to assess the antimicrobial activity of CPP extracts. 

#### 3.3.3. Hypolipidemic Effect 

Dyslipidemias are a group of asymptomatic diseases originating in abnormal concentrations of blood lipoproteins [82]. The hypolipidemic effect of the peel extract was demonstrated in a study with hamsters fed a diet containing CPP extract, and after five weeks, the plasmatic and hepatic cholesterol reduced at 35% in comparison to the control diet [83]. In a study using rats, LDL cholesterol decreased significantly through the treatment with CPP [12], owing to its high content of ascorbic acid, which protects the essential fatty acids (omega-3, omega-6, α-linolenic acid and linolenic acid) from oxidation [80,81]. Its high fiber content that helps to the lower cholesterol was also noted [11]. The CPP extract is rich in phytosterols such as lanosterol, campesteryl β-D-glucoside, stigmasteryl β-D-glucoside and sitosteryl β-D-glucoside [37], which have a hypocholesterolemic effect by a competitive mechanism with cholesterol absorption. This evidence suggests that this by-product has some potential to be employed as an ingredient or a supplement to low cholesterol and prevent cardiovascular diseases [83]. 

#### 3.3.4. Cytotoxic and Anticancer Activity

CPP extracts possess cytotoxic activity in human liver cancer cell lines (Hep G2), colorectal adenocarcinoma (Caco-2) and breast cells (MCF-7), decreasing the viability of cancer cells, by increasing the concentrations of bioactive compounds of an ethanolic extract. The highest concentrations cause a reduction in the viability of cancer cells, especially in the human liver cancer cell line (Hep G2) [84,85,86]. The anticancer effect may be due to the presence of polyphenols that play an important role in antioxidant activity and show antiproliferative activity or cytotoxicity in human cancer cells [87]. In addition, the extract contains gallic acid, which also shows cytotoxic activity against tumor cells, as in the case of lung cancer, prostate or leukemia [88]. Furthermore, sterols inhibit tumor promotion in carcinogenesis in mice, altering the expression of certain genes related to cell growth and apoptosis. Furthermore, the presence of quercetin could be one of the active compounds responsible for the anticancer and apoptosis-inducing effects of the extracts [89]. CPP contains large amounts of isorhamnetin (3’-methoxy-3,4’,5,7-tetrahydroxyflavone) that exerts anticancer action by the inhibition of epidermal growth factor (EGF); it also improves the skin barrier function through activation of the peroxisome proliferator-activated receptor (PPAR)-α and the suppression of inflammatory cytokines production. In a model of gastric cancer cells and, in combination with chemotherapeutic drugs, isorhamnetin also has strong antiproliferative effects and causes cytotoxicity [90].

#### 3.3.5. Hypoglycemic Effect

Regarding the benefits in glucose levels, in a study with a group of rats fed with CPP flour, glycaemia was lower in comparison with a group fed with flour with apple residue, and the weight gain was lower compared to the control (inulin) [91] due to the low soluble sugars, the fructans content of fructans and high fiber content in the CPP [92]. 

Different studies aim at developing nutraceuticals or dietary supplements that may provide health benefits. Although no clinical trials have been developed exclusively with CPP, the effects of the product “OpunDia^TM^”, made with cactus cladode and CPP extracts (70:30 *w/w*), has potential hypoglycemic activity. After administration of 400 mg/day for 16 weeks, in obese pre-diabetic men and women, there was a significant decrease in blood glucose concentrations in an oral glucose tolerance test [93]. Furthermore, in athletes, this dietary supplement stimulates insulin secretion before and after exercise, lowering blood glucose levels and lowering the area under the blood glucose curve by 30%, a reduction of 10% in the glucose levels and greater insulin concentrations after ingestion [94].

### 3.4. Application of CPP in Industry 

#### 3.4.1. Food Industry 

A high amount of peel waste is generated from fruit and vegetable-based industries and has led to an economical and nutritional losses. Processing of fruits and vegetables generates a significant amount of residue, among 25–30% of the total product, which have many bioactive compounds and have many applications in some industries such as food additives or ingredients, to develop films, for probiotics development, among others. The utilization of these low-cost horticultural wastes as a value-added product is a novel step for sustainable production [95], since the presence of complex polysaccharides composed of arabinose, galactose, rhamnose and galacturonic acid may influence the pleasant flavor. These characteristics make the CPP a suitable option as a sweetener in foods [12]. The great amount of carbohydrate polymers makes the peels a good source of fibers; therefore, their use is relevant to the food industry as a viscosity agent in food components [33]. 

The carbohydrates of CPP provide techno-functional properties such as water holding capacity (WHC) and lipid holding capacity (LHC), which range from 3.20 to 4.60 g/g, 1.73–1.90 g/g, respectively. Another technological property is the swelling capacity (SC), which varies from 9.82 to 12.33 mL/g. These functional properties are relevant because they may help to improve the sensory characteristics of some foods such as sausages or bakery products [37]. 

Several trials have included the CPP in different food products in order to obtain different sensorial attributes or improve nutritional value or presence of bioactive compounds. 

An assay was done adding freeze-dried CPP in a snack of rice flour produced by extrusion cooking, in order to improve the nutritional and organoleptic properties where the cooking process does not significantly affect the content of bioactive compounds [96]. In addition, a snack from CPP using instant pressure drop texturing (a new technology applying high pressure at high temperatures) was applied to develop a “healthy snack”, with a high content of phenolic compounds and β-carotene [23]. In another study, 10% CPP flour was added to a snack of amaranth and rice flour; as a result, the fat content decreased mainly due to the fiber content, which also provided better sensorial acceptance, better attributes of color, texture and oiliness [97].

CPP are used to produce biscuits rich in phenolic compounds, with greater sweetness and stable attractive colors, taking advantage of the natural colorants of the peels [98]. It has also been incorporated in the preparation of muffins mixed with wheat flour; the product presents a high fiber and moisture content and a lower fat content with great acceptability in the sensory analysis in comparison with commercial muffins [99]. 

The addition of CPP powder as a source of carbon to produce baker’s yeast, using Saccharomyces cerevisiae, has also been assayed. The maximum cell mass production is reached at 24 h of inoculum time, with a temperature of 30 °C, agitation at 200 rpm and an inoculum size of 10%. Therefore, CPP has a great potential in the production of baker’s yeast for industrial bakery applications [100].

#### 3.4.2. Animal Fodder

CPP is commonly used as animal fodder due to their nutritional properties, such as the moderate content of sugars, starch, ether extract, crude protein, amino acids, fiber, and for providing a good amount of the animal requirements for vitamins and calcium, representing a better feed for ruminants than commercial feeds [101]. In rabbits fed with diets with 50% of CPP, giblets, liver and heart were heavier, and abdominal fat, triglycerides and LDL cholesterol were reduced, while the concentration of HDL cholesterol increased [102]. Adding a 15% of CPP to the traditional corn diets for commercial Cobb chicken, the weight gain improves in 5.78%, as well as the total protein and globulin in blood serum, resulting in superior nutritional status, greater daily weight gain, and better sensorial characteristics of the meat, including taste, color, odor, texture, and general acceptability [103]. The addition of CPP to the traditional diets, lead to a greater economic efficiency by minimizing the costs of the expensive yellow corn grain which is the food base in the poultry diets [104]. Farmers consider the fruit peel as an excellent supplemental feed and usually offered animals such as draught oxen, pregnant and milking animals [105].

#### 3.4.3. Colorants

Synthetic colorants have been used in different types of industries because they present good stability and are cheap. However, the trend for using natural colorants is increasing, and the market derived from natural sources such as fruits, vegetables, insects or minerals represents a promising industry [106]. The peels of the cactus pear, mainly the red ones, are an important source of betanins, one of the most valued red natural colorants [107]. These pigments are of great importance in the industry because of their ecological value and non-toxicity. Betalains from CPP are colorants with a potential to be applied in functional foods, not only for their action as colorants also for their as antioxidants, antimicrobial, anti-proliferative and hypolipidemic properties [37,61], and are considered a permitted colorant for foods (USDA) [60].

Cactus pear by-products can be used in a more profitable way, by extracting the colorants before being used for animal feed [108], either using solvents, or novel clean technologies, such as the application of microwaves or pulsed electrical fields or ultrasound, which provide better recovery of the intracellular compounds with less impurity [107,109]. The textile fibers dyed with the extract of *Opuntia* spp. peels yield pink colors with great solidity, and by adding lemon juice (widely used in popular tradition as a natural mordant), depth tones are reached, achieving an environmentally friendly staining process [108]. 

#### 3.4.4. Other Applications 

The aqueous extract and the powder of CPP have been added in biofilms to improve the physical and antioxidant characteristics of edible carboxymethylcellulose films, obtaining a formulation with a high content of betalains and phenolic compounds by adding 1.7% of peel powder and 3.3% of the aqueous extract [110]. In addition, the mucilage from CPP is extracted to create a biopolymer with good solubility in water, foam and emulsion capacity and with a thermal stability of up to 250 °C, which could see this biopolymer applied in biodegradable containers [111]. The raw CPP has been successfully used as an agent for the decontamination of wastewaters that contain dyes, pesticides, high levels of turbidity, chemical oxygen demand and heavy metal ions. By using only 0.5 g of CPP at a particle size of 10 mm, it has highly efficient decontamination power, produced by the high biosorption capacity of the CPP [25]. 

## 4. Conclusions

The functional benefits and bio-active compounds that CPP provides generate great scientific interest in the exploration of this by-product as a inexpensive source of fibers, antioxidants, fatty acids and colorants, in addition to the proven health benefits, such as its hypolipidemic, hypocholesterolemic and cytotoxic activity. In this sense, cactus pear residues may be used on a wide spectrum of applications either as an ingredient for functional foods, as food supplements or to improve the sensorial characteristics of food and pharmaceutical products with the use of natural ingredients, which is generally better accepted in contrast to synthetic colorants or other additives. There remains great potential for future studies on the recovery of the compounds and their utilization. The health benefits that cactus pear peel could provide needs deeper research, mainly through clinical studies, in order to apply the current research. Furthermore, the use and exploitation of the by-products leads to more sustainable and environmentally friendly processes.

## Figures and Tables

**Table 1:** Nutritional composition of cactus pear peel.

	Ranges	Refs.
**Moisture (%)**	80.17–90.33	[12,37]
**Ash (%)**	1.60–3.05	[12,37]
Minerals		
Mg (mg/100 g)	0.987	
Ca (mg/100 g)	0.951	
Na (mg/100 g)	0.925	
K (mg/100 g)	0.320	[12]
Fe (mg/100 g)	0.129	
Mn (mg/100 g)	0.090	
Zn (mg/100 g)	0.090	
**Protein (%)**	0.90–4.14	[12,35]
**Lipids (%)**	0.94–2.43	[4,11]
Palmitic (%)	23.71	
Stearic (%)	3.93	
Arachidonic (%)	5.52	
Palmitoleic (%)	2.46	[12]
Oleic (%)	19.73	
Linoleic (%)	28.96	
Linolenic (%)	15.68	
**Carbohydrates**	27.60	[4]
Total sugars (%)	3.53	[35]
Reducing sugars (%)	2.07	[35]
Saccharose (mg/100 g)	0.00225	[37]
Glucose (mg/100 g)	0.014	[37]
Fructose (mg/100 g)	0.0029	[37]
Galacturonic acid (mg/100 g)	2.23	[12]
Stachyose (mg/100 g)	1.81	[12]
Mannitol (mg/100 g)	1.48	[12]
Sorbitol (mg/100 g)	0.71	[12]
Arabinose (mg/100 g)	0.05	[12]
Starch (mg/100 g)	7.12	[4]
**Fiber**		
Total fiber	40.80	[4]
Crude fiber (%)	0.96	[12]
Insoluble fiber (%)	7.98–8.12	[38]
Soluble fiber (%)	19.39–34.95	[38]
Hemicellulose (%)	20.80	[4]
Cellulose (%)	27.0	[11]
Lignin (%)	2.4	[11]
Pectin (%)	7.71	[4]
Mucilage (%)	4.10	[11]
**Vitamins**		
Ascorbic acid (mg/100 g)	27.3	
Niacin (B_3_) (mg/100 g)	0.26	[38]
Pyridoxine (B_6_) (mg/100 g)	0.19	
Folic acid (B_9_) (mg/100 g)	0.11	

**Table 2:** Bioactive compounds and antioxidant activity of cactus pear peels.

Bioactive Compounds and Antioxidant Activity	Ranges	Refs.
Phenolics (mg GAE/100 g)	14–376	[50,57]
Flavonoids (mg RE/100 g)	8–66	[57]
Tannins (mg CE/100 g)	23–144	[57]
Carotenoids (μg/g)	1.79–6.06	[50]
Betaxanthins (mg/100 g)	83.4	[53]
Betacyanins (mg/100 g)	13,468	[53]
DPPH (%)	90.9−96.8	[50]
ABTS (µM TE/100 g)	529	[50]
Chelating activity (%)	69–97	[50]
Reducing power (EC 50 mg/mL)	2.08–2.65	[57]
ORAC (µM TE/100 g)	37.4	[53]
B-Carotene bleaching inhibition (EC 50 mg/mL)	3.87–6.49	[57]

GAE: Gallic Acid Equivalent; RE: Rutin Equivalent; CE: Catechin Equivalent; TE: Trolox Equivalent; EC: Extinction coefficient.

## Data Availability

Not applicable.
